# Body mass index, performance on activities of daily living and cognition: analysis in two different populations

**DOI:** 10.1186/s12877-021-02127-8

**Published:** 2021-03-12

**Authors:** Miguel Germán Borda, Luis Carlos Venegas-Sanabria, Elkin Garcia-Cifuentes, Ronald Camilo Gomez, Carlos Alberto Cano-Gutierrez, Diego Alejandro Tovar-Rios, Vera Aarsland, Khadija Khalifa, Alberto Jaramillo-Jimenez, Dag Aarsland, Hogne Soennesyn

**Affiliations:** 1 Centre for Age-Related Medicine (SESAM), Stavanger University Hospital, PB 8100, N-4068 Stavanger, Norway; 2 Faculty of Health Sciences, University of Stavanger, Stavanger, Norway; 3 Semillero de Neurociencias y Envejecimiento, Ageing Institute, Medical School, Pontificia Universidad Javeriana, Bogotá, Colombia; 4 Unidad Geriatría Hospital Universitario San Ignacio, Bogotá, Colombia; 5 Universidad Del Valle, School of Statistics, Faculty of engineering, Santiago De Cali, Valle Del Cauca Colombia; 6 Department of mathematics and statistics, Universidad Autónoma de Occidente, Faculty of Basic Sciences, Santiago de Cali, Colombia; 7 Grupo de Neurociencias de Antioquia, School of Medicine, Universidad de Antioquia, Medellín, Colombia; 8 Grupo Neuropsicología y Conducta, School of Medicine, Universidad de Antioquia, Medellín, Colombia; 9 Department of Old Age Psychiatry, Institute of Psychiatry, Psychology, and Neuroscience, King’s College London, London, UK

**Keywords:** Obesity, Cognitive impairment, Dependency, BMI, 80 and over Aged

## Abstract

**Background:**

With this study, we aim to determine the associations of the different categories of the body mass index (BMI) with activities of daily living (ADL) and cognitive performance in two different populations living in the community; Colombian and South Korean older adults.

**Methods:**

We performed a cross-sectional analysis of two surveys separately; The Survey on Health, Well-Being, and Aging in Colombia (SABE) (*n* = 23,343) and the Korean Longitudinal Study of aging (KLoSA) (*n* = 4556). Participants older than 50 years were selected from rural and urban areas achieving a representative sample. Here we investigated the association between BMI categories with function using zero-inflated negative binomial regressions, and with cognition using logistic regression models.

**Results:**

After adjustment, in Colombia, underweight was associated with an impaired score on the Mini-mental State Examination (MMSE) and worse performance in the instrumental activities of daily living (IADL). Also, being overweight was associated with a better score on the MMSE and the IADL. For both outcomes education level significantly influenced the predictions. In South Korea, there were no significant associations for cognition, IADL, or basic activities of daily living (BADL).

**Conclusions:**

In the Colombian population, underweight, was associated with reduced cognitive performance and daily functioning. Additionally, being overweight but not obese was associated with better cognition and daily functioning. In South Korea, there were no significant associations between BMI and cognition, IADL, or BADL.

## Introduction

Due to health and social advances, older adults are living longer, and the population older than 60 is increasing [1]. This is one of the greatest human achievements, however to keep older adults living longer, but also healthy and free of dependency, presents several challenges to societies [2]. Functional deterioration in older adults has become more relevant, as a major public health problem, since functional decline is a common endpoint of multiple health conditions and is directly associated with frailty, poor quality of life, dependency, and high costs to health systems [3]. Thus, independence in activities of daily living (ADL) is an important marker of health in older adults. Autonomy is the ability of a person to satisfy their needs, independently and satisfactorily. Being autonomous requires adequate mental and physical abilities. Cognition is a central component of older adult’s health; impaired cognition is a very important risk factor for dependence and poor quality of life [4, 5]. Therefore, maintaining good cognition and performance in ADL should be priorities in health systems [6].

Besides, maintaining an adequate nutritional status has been pointed out as an important intervention to preserve well-being and health in older adults, and we and others have related nutrition with cognition and function [4, 6, 7]. Studies in younger populations have generally shown that overweight and obesity predispose to unfavorable conditions such as cardiovascular diseases, mobility issues, depression, anxiety, dementia, poor quality of life, and death [8]. Thresholds for the Body mass index (BMI) adult-categories proposed by the World Health Organization (WHO) were originally chosen because of increased all-cause mortality in those with high BMI [9, 10]. However, recent studies show that this may be different in older populations, where undernutrition is the main factor associated with negative outcomes. Interestingly, evidence has pointed out that a “U-shaped” relationship exists between nutritional status and unfavorable outcomes, where the people in the extremes (i.e. underweight and obesity) represent the groups with a higher risk of morbimortality [9, 10]. Similar associations have been found for frequent geriatric conditions such as frailty [11].

However, there is a lack of evidence regarding the association between ideal weight according to the widely used WHO BMI adult-categories and the performance in cognition and especially in ADL. Of note, this association may vary depending on factors such as genetics, diet, culture, and social and economic resources.

In Asia, South Korea as a developed country and in Latin-America, Colombia as an emerging market economy, are countries with different diets, cultures, and social and educational opportunities, but little is known about the association of the Body mass index categories with cognition and daily functioning. Thus, we aimed to evaluate the associations of the traditional BMI categories with ADL and cognitive performance in older adults living in the community in these two countries.

## Materials and methods

### Setting and participants

This is a secondary analysis of two national studies: The Health, Well-Being, and Aging/ Salud, Bienestar y Envejecimiento (SABE) Colombia study, and the Korean Longitudinal Study of Aging (KLoSA). The studies were designed to determine the factors that characterize aging in these countries.

In both studies, face-to-face interviews were conducted, and subjects were given sets of questionnaires, concerning sociodemographic characteristics, health-related issues, lifestyle habits, and cognitive function. Participants with incomplete data or those who refused follow-up could not be included in the analysis.

SABE was performed in 2015 with a representative sample of community-dwelling Colombian older adults (age ≥ 60 years). As of 2020, this is the largest database available regarding Latin American older adults. A set of questionnaires on different topics (socio-demographic characteristics, health-related issues, access to health services, cognitive performance, functional status, and financial resources) was applied to all the participants by interviewers at the older adult’s household. The effective national response rate was 66%. Complete methodology, processes, and objectives are available elsewhere [12].

**Fig. 1:**
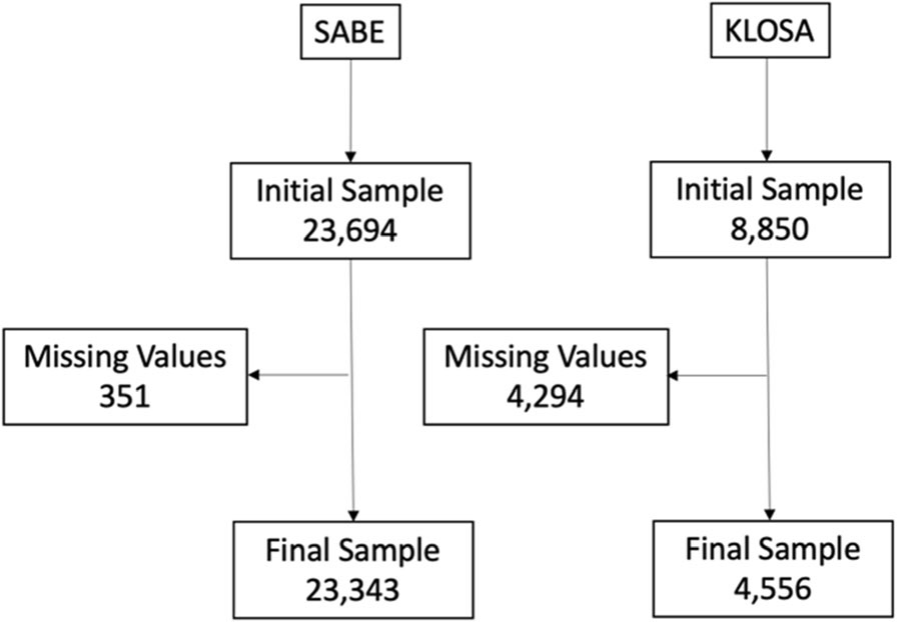
Flowchart of the study sample; SABE and KLoSA

KLoSA started in 2006, with follow-up every 2 years. A stratified multistage probability sampling was used to obtain a representative sample. The analysis was made on wave 2016. The recruitment of participants and methods used in KLoSA have been described in detail elsewhere [13, 14]. See the flowchart of the total population in Fig. 1.

### Variables

In Colombia, BMI was determined by anthropometrical data. Bodyweight and height were measured with the patient wearing light indoor clothing, using a Kendall graduated platform scale and a SECA 213® stadiometer (Hamburg, Germany), and BMI was calculated using the formula BMI = weight (kg)/height^2^ (m^2^). Four BMI categories were determined: BMI < 18.5 kg/m^2^ (underweight), BMI 18.5–24.9 kg/m^2^ (normal), BMI 25–29.9 kg/m^2^ (overweight), BMI ≥ 30 kg/m^2^ (obese), following WHO recommendations [15, 16]. In Korea, BMI was measured using self-reports of height and weight (unit: kg/m2). BMI subgroups were based on the Asia-Pacific BMI classification and four categories were determined: BMI < 18.5 kg/m^2^ (underweight), BMI 18.5 - < 23.0 kg/ m^2^ (healthy weight), BMI 23.0 - < 25.0 kg/m^2^ (overweight), and BMI ≥ 25.0 kg/m^2^ (obese) [15].

The basic activities of daily living (BADL) refer to self-care and mobility, and their deterioration is closely related to clinical complications, geriatric syndromes, frailty, and dependency [17]. In SABE, BADL were assessed using the Barthel scale (range 0–100) [18]. The KLoSA adapted existing BADL and IADL instruments to assess the functional status of the community-dwelling adult population. The KLoSA consists of 7 BADL items, including dressing, washing the face, bathing, eating, getting out of bed, toileting, and bladder/bowel management. All BADL variables from KloSA were dichotomized (not need any help = 0 and any kind of help =1) and summed [19, 20].

The instrumental activities of daily living (IADL) refer to the individual’s ability to carry out actions that link the person to the environment, allowing the use of community resources to supply their own needs.

In SABE, IADLs were assessed through the question: ‘Can you perform the following activities?’: 1) Able to manage own finances, 2) Capable of making daily purchases (especially food) 3) Able to prepare food, 4) Able to manage own medications, 5) Use of public transportation or taxi, 6) Telephone use. Answer options were codified as binary: 0. capable of performing the tasks alone (including both those who perform the activities alone without any difficulties and those who perform the activities alone with difficulty); and 1. not able to do it alone (including both those who perform the activities with any kind of help and those who cannot perform the activity) [12].

In KLoSA, 10 IADL items were evaluated, including grooming, housekeeping, preparing meals, laundering, going out, using public transportation, shopping, money management, phone use, and medication management. IADLs were further codified as 0 if the individual was able to perform the task alone (either with or without difficulty) and as 1 if he/ she was not able to perform the task by her−/himself.

A summary score was created, ranging between 0 and 6 for SABE and 0 to 10 for KLosa; a higher score reflected a greater impairment in IADL. For the IADL analysis, persons with any problems in BADLs were excluded, this to assess only those that have problems in IADL and have not progressed to having limitations on BADL [21].

In SABE, the Mini-Mental State Examination test (MMSE) in its validated Spanish version was used to determine the cognitive status (score ranging from 0 to 30). KLoSA subjects were screened using the Korean Mini-Mental State Examination (K-MMSE). The K-MMSE is a validated measure with a score ranging from 0 to 30. For both populations, the MMSE was dichotomized as normal (> 24) and cognitive impairment (≤ 24) [22–24].

As confounding variables, we included sociodemographic factors (age, sex, and years of schooling), smoking status (never, former or current smoker), involuntary weight loss (loss of more than 5 kg in the past 12 months), marital status (dichotomized as currently married/living with a partner, or alone/separated/widowed/divorced), exercise or physical activity (SABE: defined with the yes/no question *“do you perform exercise or physical activity (sports, walks ...)?”;* KloSA: people were considered physically inactive if they did < 150 min of exercise per week, and defined as physically active if they reported more than 150 min per week) and comorbidities evaluated creating a summary score, summing up each disease that was registered in the surveys by self-report (i.e. hypertension, diabetes, COPD, stroke, myocardial infarction, arthritis, and cancer), as evidence suggests that multi-morbidity accounts more efficiently for the impact on global health in older adults than individual entities [25].

### Statistical analysis

The descriptive analyses were performed by estimating percentages for categorical variables, and means and standard deviations for quantitative variables, and groups were compared using the Pearson chi-square test and t-student test, respectively. For BADL and IADL scores, we fitted a zero-inflated negative binomial regression model to evaluate the differences between nutritional levels, due to the high frequencies of zeros in the datasets (i.e. subjects without decline on BADLs or IADLs). The Barthel test score in the SABE dataset was inverted to obtain positive skewness and allow the estimation with a zero-inflated negative binomial regression. In addition, we fitted a logistic model for the MMSE score dichotomizing its values to normal cognition (> 24) and cognitive decline (<= 24). Based on the literature review we fitted two models for the following covariates: Model 1: sex, age, level of education, number of comorbidities, and Model 2: sex, level of education, number of comorbidities, smoking status, weight loss, physical activity, marital status, and age. We considered significance at *P* < .05 to evaluate the variables in the model. R software was used to perform all statistical analyses.

## Results

The final sample from Colombia was *n* = 23,343 and from Korea *n* = 4556. For Colombia, the mean ± SD of the MMSE was 23.11 ± 7.73, for IADL 0.87 ± 1.55, and BADL 96.61 ± 10.50; for Korea the MMSE 25.58 ± 5.30, 0.61 ± 2.00, BADL 0.19 ± 1.02. In both samples, there were significant differences concerning sex, number of comorbidities, marital status, smoking, exercise, weight loss, and education within BMI categories. More detailed characteristics of the samples and bivariate information see Table 1.

**Table 1:** Description of the variables according to Body mass index categories. Colombian and South Korean population

n (%) or mean ± SD	Body Mass Index classification					
	Underweight	Normal	Overweight	Obese	Total	***P*** value
***Korea (n = 4556)***						
Sex						<.001
*Female*	91 (55.83)	1101 (55.58)	638 (49.53)	663 (58.99)	2493 (54.72)	
*Male*	72 (44.17)	880 (44.42)	650 (50.47)	461 (41.01)	2063 (45.28)	
Age	76.40 ± 9.82	70.39 ± 9.72	68.98 ± 9.17	68.40 ± 8.88	69.96 ± 9.47	<.001
Comorbidity	1.18 ± 1.02	1.02 ± 1.08	1.11 ± 1.10	1.48 ± 1.19	1.16 ± 1.12	<.001
Education						<.001
*No Education*	27 (16.56)	135 (6.81)	59 (4.58)	51 (4.54)	272 (5.97)	
*< High school*	79 (48.47)	772 (38.97)	436 (33.85)	491 (43.68)	1778 (39.03)	
*High school*	39 (23.93)	760 (38.36)	579 (44.95)	417 (37.99)	1805 (39.62)	
*> High school*	18 (11.04)	314 (15.85)	214 (16.61)	155 (13.79)	701 (15.39)	
Smoke						0.044
*Non-smoker*	105 (64.42)	1336 (67.44)	840 (65.22)	792 (70.46)	3073 (67.45)	
*Past-smoker*	45 (27.61)	419 (21.15)	301 (23.37)	227 (20.2)	992 (21.77)	
*Current-smoker*	13 (7.98)	226 (11.41)	147 (11.41)	105 (9.34)	491 (10.78)	
Exercise						0.005
*No*	117 (71.78)	1188 (59.97)	745 (57.84)	691 (61.48)	2741 (60.16)	
*Yes*	46 (28.22)	793 (40.03)	543 (42.16)	433 (38.52)	1815 (39.84)	
Marital status						<.001
*Separated, widowed or single*	100 (61.35)	1531 (77.28)	1029 (79.89)	849 (75.53)	3509 (77.02)	
*Married or living with*	63 (38.65)	450 (22.72)	259 (20.11)	275 (24.47)	1047 (22.98)	
Weight status						<.001
*No changes*	114 (69.94)	1663 (83.95)	1131 (87.81)	1013 (90.12)	3921 (86.06)	
*Lost*	49 (30.06)	318 (16.05)	157 (12.19)	111 (9.88)	635 (13.94)	
BADL	0.68 ± 1.92	0.19 ± 0.99	0.14 ± 0.87	0.18 ± 0.99	0.19 ± 1.02	<.001
IADL	1.63 ± 3.35	0.64 ± 2.05	0.46 ± 1.70	0.54 ± 1.90	0.61 ± 2.00	<.001
MMSE	22.74 ± 7.20	25.45 ± 5.29	26.18 ± 4.85	25.61 ± 5.27	25.58 ± 5.30	<.001
***Colombia (n = 23,343)***						
Sex						<.001
*Female*	377 (53.55)	3450 (46.36)	4279 (55.72)	5277 (70.18)	13,383 (57.33)	
*Male*	327 (46.45)	3991 (53.64)	3400 (44.28)	2242 (29.82)	9960 (42.67)	
Age	73.10 ± 8.71	71.17 ± 8.14	69.61 ± 7.32	71.46 ± 8.86	70.81 ± 8.19	<.001
Comorbidity	0.94 ± 1.03	1.03 ± 1.05	1.30 ± 1.11	1.56 ± 1.18	1.29 ± 1.13	<.001
Education						<.001
*No Education*	230 (32.67)	2111 (28.24)	1810 (23.49)	1958 (26.91)	6113 (26.07)	
*< High school*	383 (54.40)	4075 (54.51)	4289 (55.67)	4158 (55.02)	12,907 (55.05)	
*High school*	71 (10.09)	954 (12.82)	1203 (15.67)	1056 (14.04)	3284 (14.07)	
*> High school*	20 (2.84)	330 (4.43)	401 (5.22)	382 (5.08)	1133 (4.85)	
Smoke						<.001
*Non-smoker*	233 (33.10)	3117 (41.89)	3762 (48.99)	4136 (55.01)	11,248 (48.19)	
*Past-smoker*	283 (40.20)	3163 (42.51)	3290 (42.84)	2877 (38.26)	9613 (41.18)	
*Current-smoker*	188 (26.70)	1161 (15.60)	627 (8.17)	506 (6.73)	2482 (10.63)	
Exercise						<.001
*No*	262 (37.22)	2031 (27.29)	2069 (26.94)	3330 (44.29)	7692 (32.95)	
*Yes*	442 (62.78)	5410 (72.71)	5610 (73.06)	4189 (55.71)	15,651 (67.05)	
Marital status						<.001
*Separated, widowed or single*	408 (57.95)	3523 (47.35)	3222 (41.96)	3799 (50.53)	10,952 (46.92)	
*Married or living with*	296 (42.05)	3918 (52.65)	4457 (58.04)	3720 (49.47)	12,391 (53.08)	
Weight status						<.001
*No changes*	663 (94.18)	7024 (94.40)	7307 (95.16)	7160 (95.23)	22,154 (94.91)	
*Lost*	41 (5.82)	417 (5.60)	372 (4.84)	359 (4.77)	1189 (5.09)	
BADL	96.79 ± 8.23	98.23 ± 5.68	98.48 ± 4.68	93.09 ± 16.22	96.61 ± 10.50	<.001
IADL	1.35 ± 1.81	0.82 ± 1.43	0.57 ± 1.18	1.19 ± 1.86	0.87 ± 1.55	<.001
MMSE	19.99 ± 8.90	22.90 ± 7.68	24.57 ± 6.55	22.11 ± 8.49	23.11 ± 7.73	<.001

BADL: Basic activities of daily living. IADL: Instrumental activities of daily living. MMSE: Mini-mental state Examination

### BMI associations with cognition and function

#### Colombia

**Fig. 2:**
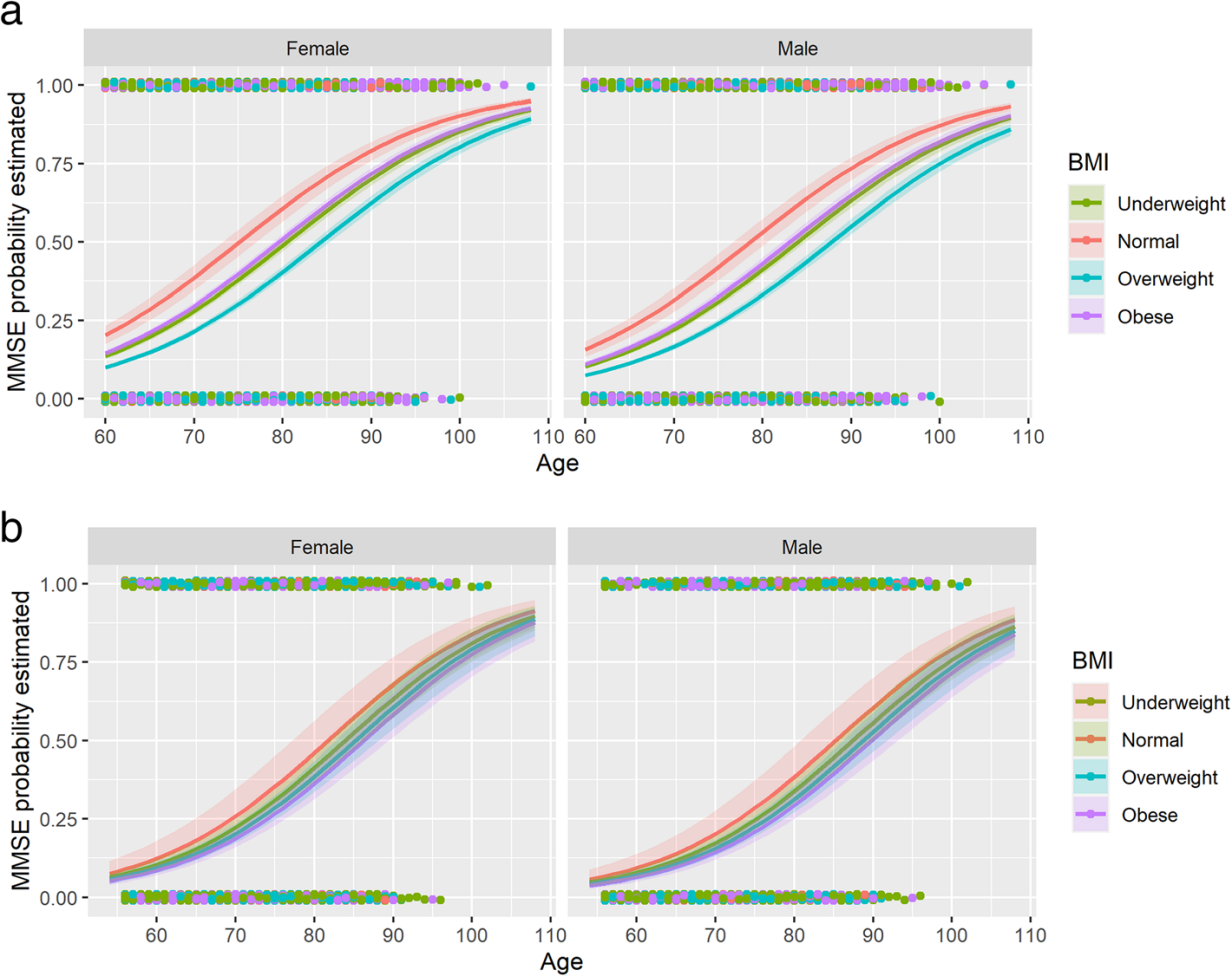
Impairment in the MMSE for the different BMI levels for (a) Colombia and (b) Korea. Higher IADL indicates more impairment. MMSE: Mini mental state examination

**Fig. 3:**
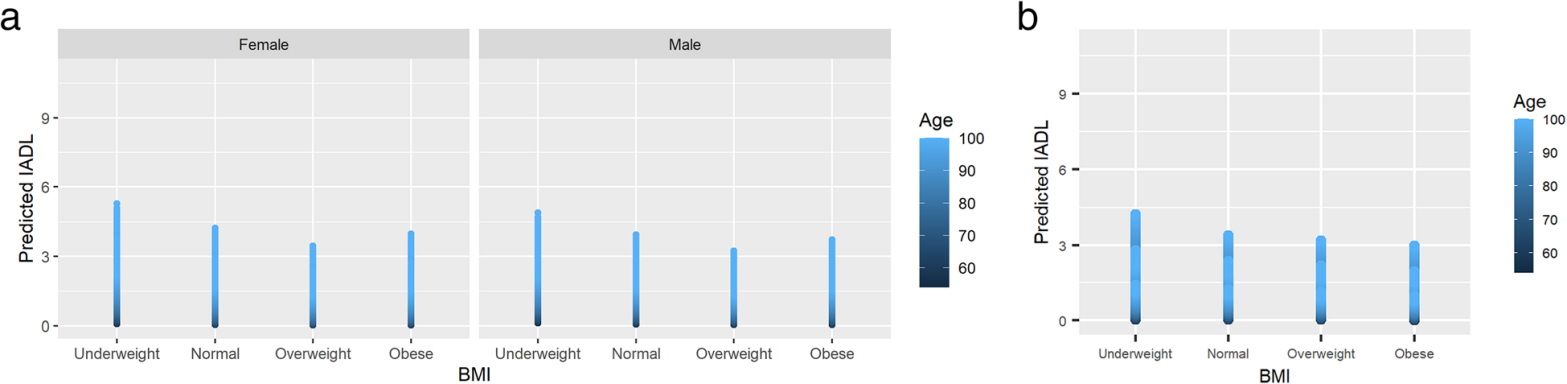
Score of IADL for the different BMI levels for (a) Colombia and (b) Korea. Higher IADL indicates more impairment. IADL: The Instrumental activities of daily living, BMI: Body mass index. Y-axis shows predicted values for IADL

**Fig. 4:**
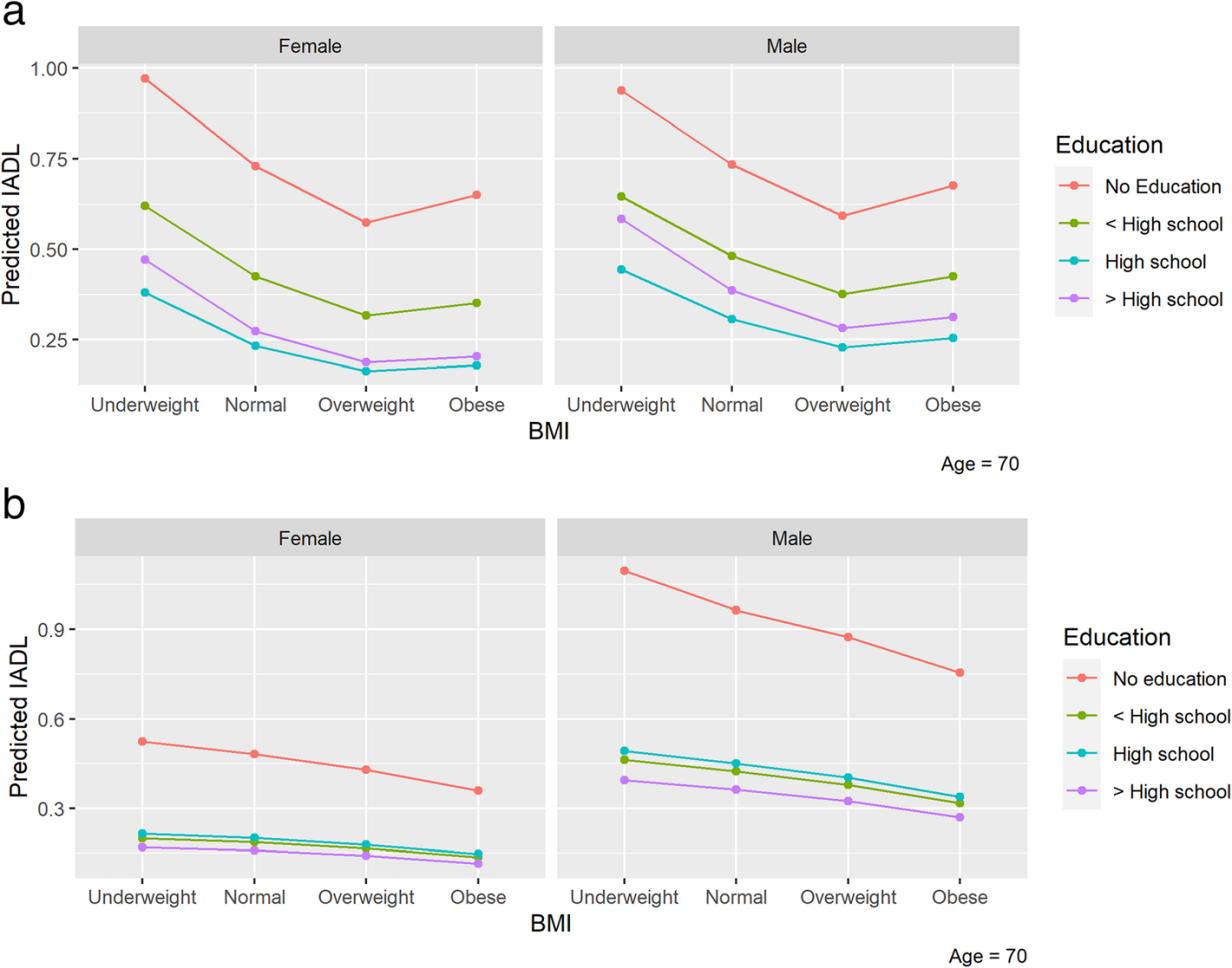
Functional status in IADL according the different BMI levels divided by level of education for (a) Colombia and (b) Korea. Higher IADL indicates more impairment. IADL: The Instrumental activities of daily living, BMI: Body mass index. Y-axis shows predicted values for IADL and BADL

- Cognition: Underweight was associated with cognitive impairment (Est 0.42, SE 0.09 *p*-value <.001.) On the other hand, being overweight was associated with normal scores in the MMSE (Est − 0.33, SE 0.04 *p*-value <.001.) Figure 2a.
- IADL: Underweight was associated with an impaired score (Est 0.17, SE 0.09 p-value 0.049.) Being overweight was associated with better scores (Est − 0.18, SE 0.05 p-value <.001.) Figure 3a. Education level had a significant confounder effect over the IADL predictions. A graphical representation of this is shown in Fig. 4a.
- BADL: Obesity (Est 0.55, SE 0.03 p-value <.001.) was associated with impaired scores. See Table 2.

**Table 2:** Association between BMI categories with function (BADL/IADL) and cognition in Colombian population

Model 1	BADL			IADL			MMSE < 24		
	Estimation	Standard Error	p value	Estimation	Standard Error	p value	Estimation	Standard Error	p value
Intercept	0.06	0.11	0.581	−3.77	0.20	**<.001**	−6.16	0.15	**<.001**
Body Mass Index									
*Underweight*	0.13	0.07	**0.047**	0.21	0.09	**0.019**	0.48	0.09	**<.001**
*Overweight*	−0.05	0.03	0.192	− 0.19	0.05	**<.001**	− 0.35	0.04	**<.001**
*Obese*	0.68	0.03	**<.001**	−0.05	0.05	0.349	0.08	0.04	0.054
Gender									
*Female*	0.12	0.03	**<.001**	− 0.09	0.04	**0.038**	−0.31	0.03	**<.001**
Education									
*< High school*	− 0.02	0.03	0.504	−0.31	0.05	**<.001**	−1.20	0.04	**<.001**
*High school*	0.00	0.04	0.984	−0.57	0.10	**<.001**	−2.32	0.07	**<.001**
*> High school*	0.16	0.07	**0.026**	−0.22	0.13	0.105	−1.78	0.10	**<.001**
Comorbidities	0.08	0.01	**<.001**	0.04	0.01	**0.005**	−0.01	0.01	0.352
Age	0.03	0.00	**<.001**	0.05	0.00	**<.001**	0.09	0.00	**<.001**
**Model 2**									
Intercept	0.79	0.11	**<.001**	−3.02	0.21	**<.001**	−4.89	0.17	**<.001**
Body Mass Index									
*Underweight*	0.07	0.06	0.288	0.17	0.09	**0.049**	0.42	0.09	**<.001**
*Overweight*	−0.03	0.03	0.424	− 0.18	0.05	**<.001**	− 0.33	0.04	**<.001**
*Obese*	0.55	0.03	**<.001**	−0.08	0.05	0.126	0.01	0.04	0.739
Gender									
*Female*	0.21	0.03	**<.001**	0.05	0.04	0.258	−0.13	0.04	**0.001**
Education									
*< High school*	−0.02	0.02	0.539	−0.31	0.04	**<.001**	−1.20	0.04	**<.001**
*High school*	0.03	0.04	0.504	−0.56	0.10	**<.001**	−2.31	0.07	**<.001**
*> High school*	0.14	0.07	**0.041**	−0.24	0.13	0.074	−1.77	0.10	**<.001**
Comorbidities	0.07	0.01	**<.001**	0.03	0.01	**0.035**	−0.02	0.02	0.147
Age	0.02	0.00	**<.001**	0.05	0.00	**<.001**	0.08	0.00	**<.001**
Smoke									
*Past-smoker*	−0.07	0.02	**0.002**	−0.11	0.03	**0.001**	−0.06	0.04	0.130
*Current-smoker*	−0.18	0.05	**<.001**	−0.03	0.05	0.496	0.21	0.06	**<.001**
Exercise									
*Yes*	−0.57	0.03	**<.001**	−0.53	0.03	**<.001**	−0.63	0.04	**<.001**
Marital status									
*Married or living with*	−0.04	0.02	0.130	−0.05	0.03	0.144	−0.24	0.04	**<.001**
Weight status									
*Lost*	− 0.09	0.05	0.080	−0.31	0.07	**<.001**	−1.03	0.09	**<.001**

#### Korea

- After adjustment for confounding variables, there were no significant associations between BMI categories with cognition, IADL, or BADL, Fig. 3b and 4b,Table 3.

**Table 3:** Association between BMI categories with function (BADL/IADL) and cognition in South Korean population

Model 1	ADL			IADL			MMSE < 24		
	Estimation	Standard Error	p value	Estimation	Standard Error	p value	Estimation	Standard Error	p value
Intercept	0.77	0.67	0.253	−0.06	0.52	0.915	−6.10	0.48	**<.001**
Body Mass Index									
*Underweight*	0.13	0.18	0.480	0.30	0.23	0.184	0.20	0.21	0.345
*Overweight*	0.09	0.14	0.545	−0.04	0.12	0.771	−0.12	0.10	0.253
*Obese*	−0.10	0.16	0.533	−0.04	0.14	0.779	−0.21	0.11	**0.049**
Gender									
*Female*	0.00	0.14	0.987	−0.03	0.14	0.813	0.32	0.09	**0.001**
Education									
*< High school*	−0.02	0.14	0.908	−0.16	0.15	0.295	−1.62	0.19	**<.001**
*High school*	0.09	0.20	0.644	−0.23	0.20	0.254	−2.42	0.21	**<.001**
*> High school*	−0.08	0.26	0.762	−0.24	0.23	0.292	−2.89	0.24	**<.001**
Comorbidities	0.00	0.05	0.940	−0.06	0.04	0.195	0.22	0.04	**<.001**
Age	0.01	0.01	0.407	0.02	0.01	**0.004**	0.09	0.01	**<.001**
**Model 2**									
Intercept	0.72	0.67	0.279	0.15	0.57	0.796	−6.12	0.51	**<.001**
Body Mass Index									
*Underweight*	0.13	0.16	0.420	0.29	0.23	0.209	0.14	0.21	0.524
*Overweight*	0.11	0.13	0.395	0.02	0.12	0.839	−0.09	0.10	0.372
*Obese*	−0.03	0.15	0.867	−0.08	0.14	0.536	−0.18	0.11	0.097
Gender									
*Female*	−0.02	0.17	0.927	−0.50	0.18	**0.004**	0.42	0.13	**0.002**
Education									
*< High school*	−0.03	0.14	0.810	−0.18	0.16	0.263	−1.58	0.19	**<.001**
*High school*	0.06	0.20	0.767	−0.37	0.22	0.091	−2.34	0.21	**<.001**
*> High school*	0.01	0.25	0.956	−0.41	0.24	0.090	−2.75	0.25	**<.001**
Comorbidities	−0.01	0.05	0.785	−0.03	0.04	0.509	0.21	0.04	**<.001**
Age	0.01	0.01	0.286	0.02	0.01	**0.019**	0.09	0.01	**<.001**
Smoke									
*Past-smoker*	0.00	0.15	0.976	−0.09	0.14	0.529	0.28	0.14	**0.045**
*Current-smoker*	−2.03	0.45	**<.001**	0.03	0.17	0.856	0.17	0.18	0.328
Exercise									
*Yes*	−0.16	0.14	0.267	0.06	0.11	0.606	−0.21	0.09	**0.019**
Marital status									
*Married or living with*	−0.17	0.14	0.228	0.38	0.14	**0.008**	0.22	0.10	**0.027**
Weight status									
*Lost*	0.24	0.12	**0.040**	0.14	0.12	0.269	0.42	0.11	**<.001**

*BADL* Basic activities of daily living. *IADL* Instrumental activities of daily living. *MMSE* Mini-mental state Examination

## Discussion

In Colombia, we found that underweight was associated with impaired MMSE score. This is in line with studies that relate malnutrition with cognitive deterioration, and have pointed out malnutrition as a risk factor for frailty and dementia [4, 26]. On the other hand, being overweight, but not obese, was associated with better MMSE. Previous studies have reported the existence of an “obesity paradox” [27]. Some studies have found that even though the risk of certain diseases increases as the BMI rises (including cardiovascular diseases, cancer, arthritis, etc.), people tend to live longer, and in older adults being a bit on the higher side of the WHO’s BMI adult-categories, appears to give an extra protective effect [28].

By contrast, in Korea, we did not find any significant associations between BMI categories, and cognition or ADL. Kim G. et al. have studied some of these associations longitudinally, reporting that those who were underweight exhibited faster deterioration in cognitive functioning, compared to those with a normal weight. However, overweight or obese older adults presented a slower cognitive decline than those with normal weight [29]. As shown in Fig. 2, the tendency of our data goes in the same direction. Even so, our study differs in many aspects. First, our study was cross-sectional, therefore, the directionality of any association could not be determined**.** Second, their study used a longitudinal model to evaluate the relationship between cognition and age, assuming that measurements were equidistant over time**.** Finally, we used K-MMSE as a dichotomic variable, because we could not fit a normal distribution for the K-MMSE score, even though we employed the usual linear transformations recommended in the literature [30]. Further research could contribute to elucidate the direction of these complex relationships more clearly, understanding the key role of potential determinants of BMI, cognitive and functional decline.

In the Colombian population, being underweight was associated with impaired performance in the IADL, whereas overweight was associated with better scores in the IADL. A possible explanation for these findings could be that when weight is lost, there is also a loss of muscle mass [31, 32] Research has shown that muscle volume protects against many kinds of negative outcomes and this might be one of the reasons why being overweight (balance between fat and muscle mass) but not obese (too much fat and low muscle mass) may protect against the unfavorable outcomes assessed in this paper [33, 34]. In addition, being underweight or lacking adequate nutrition could also affect cognitive functions and, therefore, the IADL which depend on complex cognitive processing [7, 35].

It is important to highlight that people with reduced performance on the IADL are usually at a point that can either revert or progress to having impairments in BADL. Older adults with limitations in performing BADL require more assistance from a caregiver and have more health problems related to these limitations. In this study, especially regarding IADL, WHO’s BMI adult-categories showed a U-shaped curve where extremes were related to the worst performance in IADL. This phase, when people are losing their instrumental functional capacities but still have the basic ones, might be a crucial point of intervention, where targeting nutritional status could modify functional prognosis and prevent progression to further dependency. Although this is a cross-sectional analysis, thus results might be explained by the disease(s) responsible for the functional impairment and, in second place, by the lack of mobility which is associated with both sarcopenia, physical wasting, undernutrition, and obesity [36, 37].

It worth highlighting that in both Colombia and Korea, sex and education were significant confounders for the effect of BMI over cognition and ADL. In Fig. 4, we show a graphical representation of the relevant difference between those with the highest education vs. illiterates. Here, for example, in Colombia, the worst status for those with higher education (> high school) is much better than the best status for those with low education (< high school or no education).

Education in the context of a country such as Colombia does not refer only to the knowledge acquired at school; it also refers to opportunities since early life, economic situation, and living environment. In Korea, illiterate older adults constituted 6.3% meanwhile in Colombia illiterate older adults amounted to 26%. The differences in education access and years of education in both populations represent an important disparity. Lower BMI seems to be common in those with less education. This, in low or even upper-middle-income countries such as Colombia, may connote low access to social resources, low health support, fewer opportunities, and therefore poor nutrition, and worse health-related outcomes.

In addition, we have to highlight that besides both countries having different income levels, their populations have very distinctive diets, cultures, body compositions (a normal weight person in Colombia could be classified as overweight in South Korea), and genetic backgrounds, which also could confound our results.

Furthermore, the complex relationship between undernutrition and some risk factors for morbimortality has been preliminary examined in the same population but using different datasets for both Colombia and Korea. These previously published reports have shown that underweight community-dwelling older adults have lower education, higher rates of current smoking and drinking, more comorbidities, less physical activity, poor self-rated health, and increased risks of late-life depression [38, 39]. Likewise overweight predicted decreased mortality in people living with dementia in Korean patients [40]. Therefore, specific protocols assessing longitudinally the association between BMI and cognitive or functional decline should also consider the influence of potential confounders, and analyze the direction of these relationships in order to contribute to the external validity of our preliminary findings.

This study has some limitations. First, this is a cross-sectional study; therefore, causality cannot be determined. Second, this study is based on self-reported measurements of functional status, allowing memory bias. Third, we performed a secondary analysis, which means that the variables were not collected for the specific purpose of this paper. Fourth, IADL and BADL were categorized differently and measured using different methods in the studied populations. For instance, BMI in KLoSA was determined by self-report, which may have led to recall bias. Thus, due to several differences between the measurement methods used in the surveys, we did not intend to formally compare the two cohorts and all analyses have been performed separately. Even so, we believe that presenting these results together in one paper may shed some light on a number of important issues related to the study of aging across various countries and cultures.

Besides, regarding cognition, there is still a lack of agreement concerning MMSE-thresholds for cognitive impairment that can be generalizable to heterogeneous populations in multicenter studies, as many factors can affect MMSE scores [41]. However, the cut-offs used in both populations have also been widely used in preliminary research [24, 42].

BMI is a widely used instrument, useful for important health-related outcomes that become more likely when a person is underweight or obese. Still, as with most measures of health, BMI is not a perfect measure, especially when referring to older adults; mainly due to the changes related to aging of the musculoskeletal system. Thus, a complete nutritional and anthropometric assessment would be the best way to approach body composition and nutrition in further studies. We are aware that some different BMI cut-offs are available for older populations, with adaptations according to anthropometric changes. However, problems in reproducibility using different classifications for BMI and nutritional status in older adults also have been exposed [43, 44]. Therefore, we have used the WHO’s adult cut-off points for BMI, and its validated equivalent for the Asian-Pacific population to follow the methods applied in other various large cross-sectional and longitudinal studies conducted in older persons [29, 44].

The BMI thresholds proposed by the WHO were originally chosen because an increased all-cause mortality in those with high BMI [8, 43, 45]. Nevertheless, in older adults, the relationship between BMI and mortality in several publications is not consistent [43]. In this study, a similar association was found concerning ADL and cognition as the one observed in terms of older adults’ mortality.

This study has several strengths, including data having been collected from a representative sample of old adults from two countries, one in Latin America (the biggest population-based study of adults to date), and the other in South Korea in Asia. To the best of our knowledge, there are limited reports in the literature studying the association between BMI and ADL in South America, thus this study provides new and important knowledge, as ADL in older age are associated with quality of life, wellbeing and mortality [46, 47].

## Conclusion

We found that in Colombian older adults underweight had a negative effect on both cognitive performance and daily functioning. Additionally, being overweight but not obese was associated with better cognition and daily functioning. Furthermore, the decline of function and cognition seems to be substantially associated with socioeconomic factors such as low education. This study provides relevant information for health care providers concerning future strategies for the prevention of disability.

## Data Availability

KLoSA dataset analyzed during the current study is available in an online repository: https://g2aging.org/?section=page&pageid=18 and SABE is available by request to the ministry of health of Colombia.
